# ZmOrphan94 Transcription Factor Downregulates *ZmPEPC1* Gene Expression in Maize Bundle Sheath Cells

**DOI:** 10.3389/fpls.2021.559967

**Published:** 2021-04-08

**Authors:** Alicja M. Górska, Paulo Gouveia, Ana Rita Borba, Anna Zimmermann, Tânia S. Serra, Pedro Carvalho, Tiago F. Lourenço, M. Margarida Oliveira, Christoph Peterhänsel, Nelson J. M. Saibo

**Affiliations:** ^1^Instituto de Tecnologia Química e Biológica António Xavier, Universidade Nova de Lisboa, Oeiras, Portugal; ^2^Instituto de Biologia Experimental e Tecnológica, Oeiras, Portugal; ^3^Institut für Botanik, Leibniz Universität Hannover, Hannover, Germany

**Keywords:** C4 metabolism, photosynthesis, transcriptional regulation, *cis*-elements, phosphoenolpyruvate carboxylase 1, cell-specific gene expression

## Abstract

Spatial separation of the photosynthetic reactions is a key feature of C_4_ metabolism. In most C_4_ plants, this separation requires compartmentation of photosynthetic enzymes between mesophyll (M) and bundle sheath (BS) cells. The upstream region of the gene encoding the maize PHOSPHOENOLPYRUVATE CARBOXYLASE 1 (ZmPEPC1) has been shown sufficient to drive M-specific *ZmPEPC1* gene expression. Although this region has been well characterized, to date, only few *trans*-factors involved in the *ZmPEPC1* gene regulation were identified. Here, using a yeast one-hybrid approach, we have identified three novel maize transcription factors ZmHB87, ZmCPP8, and ZmOrphan94 as binding to the *ZmPEPC1* upstream region. Bimolecular fluorescence complementation assays in maize M protoplasts unveiled that ZmOrphan94 forms homodimers and interacts with ZmCPP8 and with two other *ZmPEPC1* regulators previously reported, ZmbHLH80 and ZmbHLH90. Trans-activation assays in maize M protoplasts unveiled that ZmHB87 does not have a clear transcriptional activity, whereas ZmCPP8 and ZmOrphan94 act as activator and repressor, respectively. Moreover, we observed that ZmOrphan94 reduces the trans-activation activity of both activators ZmCPP8 and ZmbHLH90. Using the electromobility shift assay, we showed that ZmOrphan94 binds to several *cis*-elements present in the *ZmPEPC1* upstream region and one of these *cis*-elements overlaps with the ZmbHLH90 binding site. Gene expression analysis revealed that *ZmOrphan94* is preferentially expressed in the BS cells, suggesting that ZmOrphan94 is part of a transcriptional regulatory network downregulating *ZmPEPC1* transcript level in the BS cells. Based on both this and our previous work, we propose a model underpinning the importance of a regulatory mechanism within BS cells that contributes to the M-specific *ZmPEPC1* gene expression.

## Introduction

Most plants use ribulose-1,5-bisphosphate carboxylase/oxygenase (RuBisCO) as the primary carbon dioxide (CO_2_) fixing enzyme in a process called Calvin-Benson cycle. However, due to its dual activity (carboxylase and oxygenase) and the high atmospheric O_2_ concentration, RuBisCO shows a high oxygenase activity in C_3_ plants (Portis and Parry, 2007). This activity leads to the production of 2-phosphoglycolate, which is toxic for the plant and needs to be recycled through a process called photorespiration. This is a wasteful process, which leads to the loss of C, N, and ATP, thus decreasing photosynthetic efficiency (Bauwe et al., 2010). C_4_ plants, which have evolved independently from C_3_ species over 60 times (Sage, 2004), have a carbon concentrating mechanism that significantly minimizes photorespiration. To concentrate CO_2_ around RuBisCO, C_4_ plants developed a spatial separation of photosynthetic reactions between mesophyll (M) and bundle sheath (BS) cells. In M cells, carbonic anhydrase (CA) converts atmospheric CO_2_ to bicarbonate (HCO_3_^−^), which in presence of phosphoenolpyruvate (PEP) is fixed by phosphoenolpyruvate carboxylase (PEPC) to form oxaloacetate (OAA). Subsequently, OAA is rapidly converted to malate or aspartate, which diffuses into BS cells, where RuBisCO is present. Decarboxylation of these organic acids in the BS cells leads to a high CO_2_ concentration around RuBisCO, which then incorporates CO_2_ into the Calvin-Benson cycle in a highly efficient way (Furbank and Taylor, 1995).

All enzymes required for C_4_ photosynthesis are present in C_3_ species but they are low abundant and/or present in both cell types (Aubry et al., 2011). Thus, for C_4_ genes to be highly expressed and restricted to either M or BS cells, changes at *trans*- and *cis*-regulatory level had to occur (Hibberd and Covshoff, 2010; Reeves et al., 2017). A number of *cis*-elements involved in cell-specific expression of C_4_ genes have already been identified. For example, BS-specific expression of C_4_ genes has been associated with sequences within their promoter (Wiludda et al., 2012), untranslated region (Patel et al., 2006), and coding sequences (Brown et al., 2011; Reyna-Llorens et al., 2018), whereas sequences within untranslated regions (Kajala et al., 2012; Williams et al., 2016) and promoters (Stockhaus et al., 1997; Nomura et al., 2000; Gowik et al., 2016; Gupta et al., 2020) have been associated with M-specific expression.

M-specific *PEPC* gene expression has been mainly associated with regulation at promoter level. For example, in *Flaveria trinervia*, a specific promoter domain called M expression module 1 (MEM1) was reported to drive M-specific *PEPC* expression (Gowik et al., 2004). MEM1 is a 41 bp element located in a distal *ppcA* promoter region. It functions as an enhancer element, conferring M-specific gene expression, and acts as a repressor of *ppcA* expression in BS and in vascular bundle (Gowik et al., 2004; Akyildiz et al., 2007). In maize, a 0.6 kb *ZmPEPC1* upstream region was shown to be sufficient to drive M-specific gene expression (Taniguchi et al., 2000; Kausch et al., 2001). M-specific gene expression was also observed when a 1.2 kb *ZmPEPC1* upstream region driving the *β-glucuronidase* (*GUS*) reporter gene was transformed in rice, a C_3_ plant (Matsuoka et al., 1994). Although, the upstream regions involved in M-specific *ZmPEPC1* expression have been determined, the knowledge about trans-factors involved in this regulation remains scarce. The transcription factors (TFs) identified as binding to the *ZmPEPC1* upstream region include maize nuclear factors (MNFs; Yanagisawa and Izui, 1990, 1992), PEP-I (Kano-Murakami et al., 1991), DOF1 and DOF2 (Yanagisawa and Sheen, 1998), and ZmbHLH80 and ZmbHLH90 (Górska et al., 2019). Interestingly, in the *zmdof1* maize knockdown mutant, no alterations in *ZmPEPC1* gene expression were observed, suggesting a possible redundant function of TFs binding to the *ZmPEPC1* promoter (Cavalar et al., 2007). Thus, to understand the mechanism regulating the *ZmPEPC1* expression, a different approach is needed. Instead of focusing on individual TFs, it is important to investigate the transcriptional regulatory machinery involved in C_4_ *ZmPEPC1* expression. It is, therefore, important to identify novel TFs binding to the *ZmPEPC1* promoter and to determine their interactions with other TFs as well as with other proteins. The biological meaning of all these interactions must also be studied.

In this study, we aimed to identify novel maize TFs involved in the M-specific *ZmPEPC1* gene expression and to determine their function. Using a yeast one-hybrid (Y1H) system, three TFs belonging to different TF families were identified as binding to *ZmPEPC1* upstream region and functionally characterized. We show that one of the novel TFs is a BS-preferentially expressed repressor that interacts with other *ZmPEPC1* regulators and reduces their trans-activation activity. Based on our findings, we propose a model in which a new repressor together with previously identified TFs jointly contribute to the *ZmPEPC1* cell-specific gene expression.

## Materials and Methods

### Yeast One-Hybrid Screening of the Maize Leaf cDNA Expression Library

The 781 bp *ZmPEPC1* (GRMZM2G083841) upstream region (starting from ATG) was divided into three overlapping fragments (5 U, F1, and F2) and these were amplified by PCR using primers listed in Supplementary Table S3. The isolated fragments were cloned into the pINT/HIS vector system (Ouwerkerk and Meijer, 2001) and integrated into the yeast strain Y187 (Clontech, CA, United States) to originate the yeast bait strains. The yeast baits were then transformed with 1 μg of maize cDNA expression library as described by Ouwerkerk and Meijer (2001). The maize cDNA expression library used in this study was described by Borba et al. (2018). For each bait, at least 1 × 10^6^ yeast colonies were screened. The screenings were performed in complete minimal (CM)/-His/-Leu medium supplemented with: 25 mM (5 U), 10 mM (F1), and 5 mM (F2) of 3-amino-1,2,4-triazole (3-AT). Plasmids from the yeast colonies grown on the selection media were extracted, sequenced, and the obtained cDNA insert sequences were analyzed using BLAST program. The plasmids containing ZmHB87 (GRMZM26163641), ZmOrphan94 (GRMZM2G127426), and ZmCPP8 (GRMZM2G096600) and the empty vector (EV) were re-transformed into the *ZmPEPC1* yeast baits (5 U, F1, and F2). The growth of the transformed yeast was analyzed on CM/-His/-Leu medium supplemented with increasing concentration of 3-AT.

### Plant Materials and Growth Conditions

Maize plants (B73) used for M protoplast isolation, M and BS cell isolation, as well as for diurnal gene expression studies were grown as described by Górska et al. (2019).

### Isolation and Transformation of Maize and Rice Protoplasts

Maize M protoplasts were isolated from second leaves of 10-day-old maize etiolated seedlings using a modified protocol from Sheen (1991). In brief, the mid veins from the second leaves were removed and leaves were cut into approximately 0.5–1 cm strips. The strips were transferred to an enzyme solution (0.4 M mannitol, 10 mM MES pH 5.7, 1 mM CaCl_2_, 0.1% BSA, 50 mg L-1 ampicillin, 5 mM β-mercaptoethanol), 1.5% Cellulase R10 (Duchefa, Haarlem, The Netherlands), and 0.3% Macerozyme R10 (Duchefa, Haarlem, The Netherlands) and subjected to vacuum infiltration for 30 min. Afterward, digestion was continued for 5 h at 25–27°C with gentle agitation (40 rpm) in dark. After 5 h, the protoplasts were released by increasing the agitation to 85 rpm for 15 min. The enzyme solution containing protoplasts was filtered twice through a 100 μm mesh, washed with 1x volume of wash solution (154 mM NaCl, 125 mM CaCl_2_, 5 mM KCl, and 2 mM MES pH 5.7) and filtered again through a 50 μm filter. Protoplasts were harvested by centrifugation in a “swing-out” bucket (150 × *g*, 5 min) and resuspended in 200 μl MMg solution (0.4 M mannitol, 4 mM MES pH 5.7, 15 mM MgCl2). Afterward, protoplasts were diluted to a 2 × 10^6^ ml^−1^ concentration and permeabilized/transformed with polyethylene glycol (PEG) by gentle mixing 200 μl of protoplast solution with 20 μl of plasmid DNA mix and 220 μl of PEG solution (PEG 4000 40%, 0.3 M mannitol, 0.1 M CaCl_2_). After being incubated at room temperature (RT) in dark for 20 min., protoplasts were diluted with 3x volume of wash solution, harvested by centrifugation in a “swing-out” bucket (150 × *g*, 5 min) and resuspended in 750 μl of incubation solution (0.6 M mannitol, 4 mM MES pH 5.7, 4 mM KCl). After this, protoplasts were transferred to a 24-well-plate containing additional 750 μl of incubation solution with 50 mg L-1 ampicillin and incubated for 15–16 h at RT in dark.

Rice protoplasts were obtained using a protocol described by Cordeiro et al. (2016), with modifications. 2-day-old rice (cv. Nipponbare) cell suspension cultures were collected by centrifugation in a “swing-out” bucket (150 × *g*, 5 min). Collected cells were resuspended in an enzyme solution and subjected to vacuum infiltration. Following enzymatic digestion, the enzyme solution containing protoplasts was filtered through a 100 μm mesh, washed with 1x volume of wash solution and filtered through a 50 μm mesh. Protoplasts were harvested by centrifugation in a “swing-out” bucket (150 × *g*, 5 min) and resuspended in 200 μl MMg solution. These protoplasts were then diluted to a 1 × 10^6^ ml^−1^ concentration and transformed with PEG by gentle mixing 200 μl of protoplast solution with 10 μl of plasmid DNA mix and 220 μl of PEG solution. Afterwards, transformed protoplasts were incubated at RT in dark for 20 min., diluted with 3x volume of wash solution, harvested by centrifugation in a “swing-out” bucket (150 × *g*, 5 min) and resuspended in 750 μl of incubation solution (0.4 M mannitol, 4 mM MES pH 5.7, 20 mM KCl). The transformed protoplasts were incubated for 15–16 h at RT in dark.

### Bimolecular Fluorescence Complementation Assay

ZmHB87, ZmOrphan94, and ZmCPP8 full-length CDS were amplified from maize (*Zea mays* B73) cDNA by PCR, using primers listed in Supplementary Table S3. The amplified products were recombined into pDONR221 (Invitrogen, CA, United States) according to manufacturer’s instructions, confirmed by sequencing, and cloned into pYFPN43 and pYFPC43 plasmids to be in fusion with the N- and C-terminal halves of the yellow fluorescent protein (YFP), respectively. The final vectors prepared were then analyzed by digestion with restriction enzymes. Cloning of ZmbHLH80 and ZmbHLH90 TFs into pYFPC43 and pYFPN43 vectors was described by Górska et al. (2019). The resulting pYFPC43 and pYFPN43 constructs (6 μg of each plasmid) were transformed into maize M protoplasts. Each transformation was performed in triplicate. pYFPN43::ZmOrphan94 co-transformed with pYFPC::Akin3 (*Arabidopsis* SNF1 Kinase Homologue 3) and pYFPC43::ZmOrphan94 co-transformed with pYFPN43::Akin10 (*Arabidopsis* SNF1 Kinase Homologue 10) served as negative controls. The transformed protoplasts were incubated for 15–16 h at RT in dark, and reconstitution of the fluorescence signal was observed using confocal laser scanning microscopy (Leica SP5).

### Trans-Activation Assays in Maize and Rice Protoplasts

The construction of 5 U-*ZmPEPC1* and unrelated DNA sequence (US) reporter vectors, *firefly* luciferase (LUC) transformation control plasmid, as well as ZmbHLH80, ZmbHLH90, and EV effector plasmids was described by Górska et al. (2019). For F2-*ZmPEPC1* reporter plasmid, a sequence of the F2-*ZmPEPC1* fragment used in the Y1H screening was cloned (through BP-Gateway reaction) into the p2GW7m35S::GUS plasmid. For the effector constructs, the ZmHB87, ZmOrphan94, and ZmCPP8 entry clones were recombined *via* LR-Gateway reaction into p2GW7 plasmid. The trans-activations assays were carried out by transforming maize M protoplasts with 6, 6, and 16 μg of reporter, transformation control, and effector plasmids, respectively. Each transformation was performed in triplicate. The trans-activation activity of the TFs was calculated as GUS/LUC ratio. The transformed protoplasts were incubated for 15–16 h at RT in dark. Cell lysis and determination of GUS and LUC activity levels were performed as described by Figueiredo et al. (2012).

To analyze the trans-activation activity of ZmOrphan94 on 5 U-*ZmPEPC1* and mut5U-*ZmPEPC1*, we used a dual luciferase system. Thus, to construct the 5 U-*ZmPEPC1* and mut5U_*ZmPEPC1* reporter plasmids, a sequence of the 5 U-*ZmPEPC1* fragment, used in the Y1H screening, and mut5U-ZmPEPC1 (5 U-*ZmPEPC1* fragment with the ZmOrphan94 binding sites mutated from CACA to TATA), were cloned into the pGreenII 0800-LUC plasmid (Hellens et al., 2005), upstream of a minimal CaMV35S promoter driving LUC, using the restriction enzymes *Nco*I and *Spe*I. The transformation control is calculated using the activity of *renilla* luciferase (REN), which is expressed by the same vector under the control of the CaMV35S promoter. Regarding the effector, the ZmOrphan94 entry clone was recombined *via* LR-Gateway reaction into the p2GW7 plasmid. The trans-activations assays were carried out by transforming rice protoplasts with 5 μg of reporter plus 5 μg of effector plasmids. Each transformation was performed in triplicate and results shown correspond to two independent experiments. The trans-activation activity of the TFs was calculated as LUC/REN ratio. The transformed protoplasts were incubated for 15–16 h at RT in dark. Cell lysis and determination of LUC and REN activity were performed with the Dual-Luciferase® reporter assay (Promega, United States), using a modified protocol. Briefly, protoplasts were collected by centrifugation and cell lysis performed using 100 μl of 1x Passive Lysis Buffer. LUC and REN activity reactions were performed in 96-well plates using 50 μl of cell lysate, to which 30 μl of LARII reagent was added for LUC activity and 30 μl of Stop & Glo® reagent for REN activity. Luminescent was detected using FLUOStar Optima (BMG LabTech, Germany) microplate reader. Each sample was analyzed for each luciferase (LUC and REN) with measurements every 0.5 s during 12 s of luminescence acquisition.

### Production of Recombinant Trx::ZmOrphan94 Protein

The full-length CDS of ZmOrphan94 was amplified from maize (*Zea mays* B73) cDNA by PCR using primers listed in Supplementary Table S3. The amplified sequence was cloned as an *Eco*RI-*Xho*I fragment into pET32a vector (Novagen) to raise an N-terminal translational fusion with Thioredoxin (Trx) and transformed into *Escherichia coli* Rosetta (DE3) pLysS competent cells for the expression of the recombinant protein. The bacterial cells transformed with Trx::ZmOrphan94 plasmid were grown in Luria-Bertani (LB) medium at 37°C to an OD_600_ of 0.6. Subsequently, the expression of Trx::ZmOrphan94 recombinant protein was induced with 4 mM isopropyl-D-1-thiogalactopyranoside (IPTG) and continued for 16 h at 18°C. The Trx::ZmOrphan94 protein purification was performed as described by Cordeiro et al. (2016).

### Electrophoretic Mobility Shift Assay

For electrophoretic mobility shift assay (EMSA), DNA probes were generated by annealing oligonucleotide pairs and radiolabelling as described by Serra et al. (2013). Oligonucleotide sequences and respective annealing temperatures are listed in Supplementary Table S2. The binding reactions were performed in a 10 μl volume containing 1 μg of Trx::ZmOrphan94, 50 fmol of radiolabelled probe, 10 mM HEPES (pH 7.9), 40 mM KCl, 1 mM EDTA (pH 8), 1 mM DTT, 50 ng herring sperm DNA, 15 μg BSA and 10% (v/v) glycerol for 1 h on ice. The resulting complexes were resolved on a native 5% polyacrylamide gel (37.5:1). Competition assays were performed by adding 200- to 400-fold molar excess of the unlabelled probe. Trx protein was used as negative control. Gel electrophoresis and detection of radioactive signal were performed as described by Serra et al. (2013).

### Isolation of Maize Mesophyll and Bundle Sheath Cells

Mesophyll cells were isolated from the third leaves of 10-day-old maize seedlings according to Covshoff et al. (2013) with the following modification: the isolated M cells were collected to a tube containing 450 μl RLT buffer from the RNeasy Plant Mini Kit (Qiagen, Hilden, Germany). The same leaves that were used to extract M cells, were further used in BS isolation. From this point on, the isolation of BS was performed as described by Markelz et al. (2003) with the following modification: high speed shredding was carried out three times for 1 min each. Samples for cell isolation were harvested at 6 and 2 h before lights turned on (−6 h, −2 h), when the lights turned on (0 h), and 2 h after illumination (+2 h). The samples collection at time point −6 and −2 h was performed under green light. For each time point, three biological replicates of M and BS were prepared using five leaves per replicate.

### RNA Isolation, cDNA Synthesis, and Quantitative PCR

Total RNA was extracted from purified M and BS samples, and from whole maize leaves using the RNeasy Plant kit (Qiagen, Hilden, Germany). After isolation, total RNA was treated with Turbo DNase (Ambion, CA, United States) according to the manufacturer’s instructions. The quality of the RNA was assessed by NanoDrop 2000c Spectrophotometer (Thermo Fisher Scientific, MA, United States) and by gel electrophoresis. First strand cDNA was synthesized using SuperScript® III First-Strand Synthesis System (Invitrogen, CA, United States) following the manufacturer’s instructions. Two-hundred and eighty and five-hundred nanogram of total RNA from the purified cell samples and whole maize leaves, respectively, were used to synthesize cDNA using oligo (dT) primers. The quantitative PCR (qPCR) was performed using SYBR Green I Master mix (Roche, Basel, Switzerland) on a LightCycler 480 system (Roche, Basel, Switzerland). Threshold cycles (Ct) values were calculated from means of three biological replicates, three technical replicates each. The Ct values were normalized against *ZmActin*1 (GRMZM2G126010) for diurnal analysis, and GRMZM2G144843 and GRMZM2G044552 for cell-specific analysis. GRMZM2G144843 and GRMZM2G044552 were selected based on their stable expression between M and BS cells and along the time (data not shown). Gene specific primers used in qPCR are listed in Supplementary Table S3.

### Direct Yeast One-Hybrid

The full-length coding sequence of *OsOrphan65* was amplified from rice (*Oryza sativa* L, cv. Nipponbare) cDNA using the primers listed in Supplementary Table S3. The amplified product was recombined in pDONR221 (Invitrogen, CA, United States), confirmed by sequencing and cloned into the pDEST22 (Invitrogen, CA, United States) plasmid, according to manufacturer’s instructions. Integrity of the expression clone was analyzed by digestion with restriction enzymes. For the direct yeast Y1H, OsOrphan65::pDEST22, ZmbHLH90::pDEST22 (positive control), and pDEST22 (negative control) were individually transformed into the yeast bait strains containing 5 U, F1, and F2 *ZmPEPC1* upstream fragments. The growth of the transformed yeast baits was analyzed on CM/-His/-Trp medium and with increasing concentrations of 3-AT.

## Results

### ZmHB87, ZmCPP8, and ZmOrphan94 Bind to the *ZmPEPC1* Upstream Region

**Figure 1:**
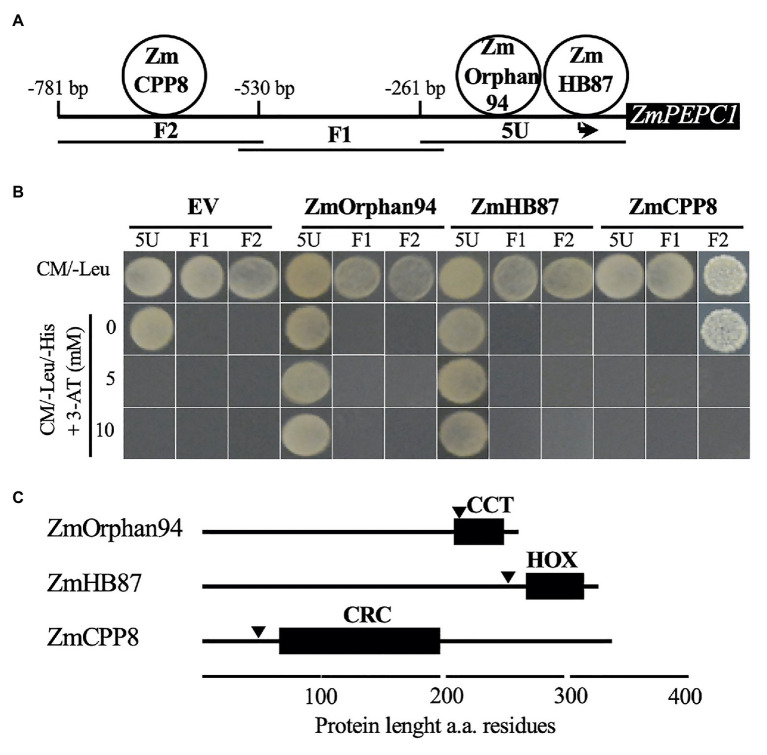
Analysis of the transcription factors (TFs) binding to *ZmPEPC1* upstream region. **(A)** Schematic representation of the three *ZmPEPC1* upstream region fragments used in the yeast one-hybrid screenings. ZmHB87 and ZmOrphan94 were identified as binding to the 5 U fragment located between −261 and 0 bp upstream of *ZmPEPC1* ATG. The order of the TFs binding to 5 U fragment is representative but was not determined experimentally. ZmCPP8 was identified as binding to the F2 fragment located between −530 and −781 bp upstream of *ZmPEPC1* ATG. **(B)** Validation of the interactions between identified TFs and *ZmPEPC1* upstream region. Yeast bait strains carrying the different fragments of the *ZmPEPC1* upstream region were transformed with plasmids to express ZmHB87, ZmOrphan94, and ZmCPP8, as well as with empty vector (EV). Growth of the transformed bait strains was analyzed on complete minimal (CM)/-Leu/-His medium (CM medium lacking Leucine and Histidine) supplemented with increasing concentrations of 3-amino-1,2,4-triazole (3-AT). **(C)** Schematic representation of ZmHB87, ZmOrphan94, and ZmCPP8 protein structures with homeodomain (HOX), CONSTANS, CO-like, and TOC1 (CCT), and C1-RNPXAFXPK-C2 (CRC) DNA-binding domains, respectively, determined by ScanProsite. Nuclear localization signals (arrowheads) were predicted by cNLS mapper. a.a. refers to amino acid.

To identify additional components of the *ZmPEPC1* regulatory network and isolate TFs involved in the M-specific *ZmPEPC1* gene expression, we used a Y1H approach to screen a maize cDNA expression library. The Y1H screening was carried out using overlapping fragments of the *ZmPEPC1* upstream region (5 U, F1, and F2; Figure 1A) as baits. These fragments were cloned upstream of the *HIS3* reporter gene and the resulting constructs were individually integrated into yeast genome to generate 5 U-*ZmPEPC1*, F1-*ZmPEPC1*, and F2-*ZmPEPC1* yeast bait strains, respectively. These baits were transformed with the maize cDNA expression library and the yeast growth on CM/-Leu/-His selection medium supplemented with 3-amino-1,2,4-triazole (3-AT), a competitive inhibitor of the *HIS3* gene product, was analyzed. Among the colonies that grew on the selection media, we identified three different TFs, ZmCPP8, ZmHB87, and ZmOrphan94. ZmHB87 and ZmOrphan94 were identified as binding to 5 U fragment, whereas ZmCPP8 was found to interact with F2 (Figure 1A). No TFs were identified as binding to the F1 fragment. To validate the TF-DNA interactions and determine their specificity, we isolated the plasmids and re-transformed each of the *ZmPEPC1* baits (5 U-*ZmPEPC1*, F1-*ZmPEPC1*, and F2-*ZmPEPC1*) with the plasmids expressing the identified TFs and with the EV as negative control. The growth of the re-transformed bait strains was analyzed on CM/-Leu/-His media supplemented with increasing concentrations of 3-AT. According to our results, ZmHB87 and ZmOrphan94 bind specifically to the 5 U fragment (Figure 1B). Though the 5 U-*ZmPEPC1* bait strain transformed with the EV grew on the CM/-Leu/-His selection medium without 3-AT, the presence of 5 mM 3-AT was enough to abolish yeast growth. The same bait strain transformed with the plasmids expressing ZmHB87 or ZmOrphan94 grew on the CM/-Leu/-His selection medium supplemented with up to 10 mM 3-AT (Figure 1B), showing the authenticity of their protein-DNA interactions. Regarding ZmCPP8, our results indicate that it binds specifically to the F2 fragment. When the F2-*ZmPEPC1* bait was transformed with the EV, it did not grow on CM/-Leu/-His, but it grew when transformed to express ZmCPP8 (Figure 1B). However, our results suggest that the interaction between ZmCPP8 and the F2 fragment is not very strong as the yeast growth is eliminated with 5 mM 3-AT.

*In silico* analysis of ZmCPP8, ZmHB87, and ZmOrphan94 protein sequences revealed that all three TFs contain DNA-binding domains and nuclear localization signals, supporting their role as transcriptional regulators (Figure 1C, Supplementary Figure S1A).

### ZmOrphan94 Forms Homodimers and Interacts With Other TFs Binding to the *ZmPEPC1* Upstream Region

**Figure 2:**
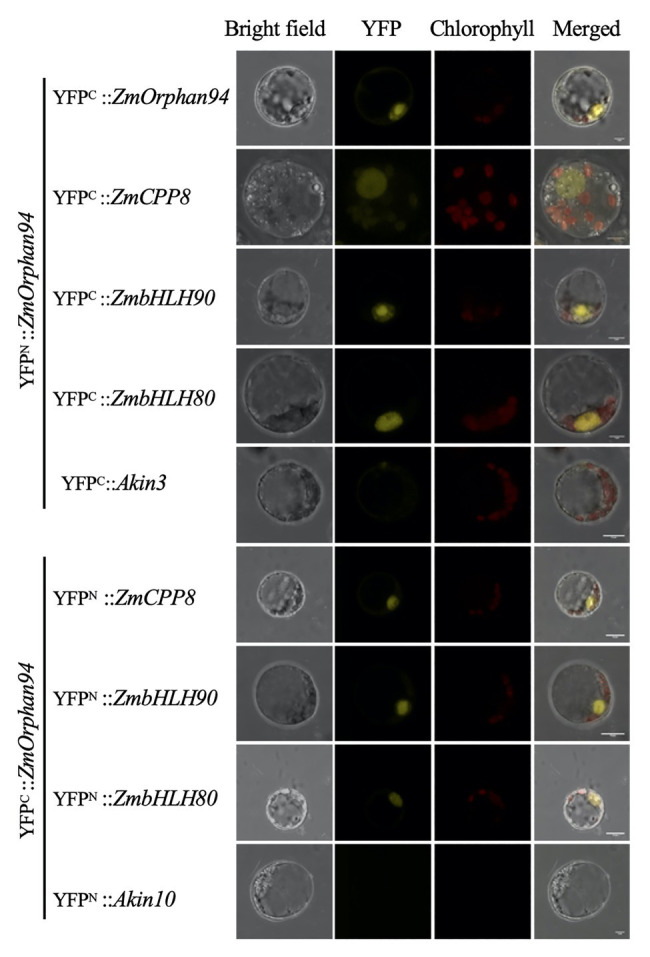
Bimolecular fluorescence complementation (BiFC) analysis of protein-protein interactions between OsOrphan94 and other TF binding to *ZmPEPC1* upstream region. Pairs of proteins fused to complementary yellow fluorescent protein (YFP) halves were transiently expressed in etiolated maize mesophyll protoplasts. BiFC fluorescence is indicated as the YFP signal. Maize mesophyll protoplasts co-transformed with YFP^C^::*Akin3* and YFP^N^::*ZmOrphan94*, and YFP^N^::*Akin10* with YFP^C^::*ZmOrphan94* were used as negative controls. Scale bars = 10 μm.

Given that protein-protein interactions are an important feature influencing TF activity, we decided to investigate whether ZmCPP8, ZmHB87, and ZmOrphan94 form homodimers and/or heterodimers. To test this, all TFs were cloned into pYFN43 and pYFC43 vectors to raise N-terminal translational fusions with N- and C-halves of the YFP and then used to perform bimolecular fluorescent complementation (BiFC) assays. These assays were carried out in maize M protoplasts and all possible interactions were tested. According to our results, among the novel TFs, only ZmOrphan94 forms homodimers. A strong fluorescent signal was observed when YFP^N^::*ZmOrphan94* was co-transformed with YFP^C^::*ZmOrphan94* (Figure 2), but no fluorescence was detected in protoplasts co-transformed with YFP^N^::*ZmCPP8* and YFP^C^::*ZmCPP8*, nor with YFP^N^::*ZmHB87* and YFP^C^::*ZmHB87* (data not shown). Regarding the interactions between the novel TFs, our results show that ZmOrphan94 interacts with ZmCPP8 (Figure 2). An YFP fluorescent signal was detected in the nuclei of protoplasts co-transformed with YFP^N^::*ZmOrphan94* and YFP^C^::*ZmCPP8*, as well as with YFP^C^::*ZmOrphan94* and YFP^N^::*ZmCPP8*. No interactions were observed between ZmOrphan94 and ZmHB87, neither between ZmHB87 and ZmCPP8 (data not shown).

Our previous studies have identified two ZmbHLH TFs, ZmbHLH80 and ZmbHLH90, as binding to the *ZmPEPC1* upstream region (Górska et al., 2019). Thus, to gain deeper insights into the *ZmPEPC1* regulatory network, we decided to analyze whether the novel TFs could interact with these ZmbHLHs. Interestingly, we found that ZmOrphan94 interacts with either ZmbHLH80 or ZmbHLH90. Reconstitution of the YFP signal was observed in nuclei of protoplasts co-transformed with YFP^N^::*ZmOrphan94* and YFP^C^::*ZmbHLH90* or YFP^C^::*ZmbHLH80*, and YFP^C^::*ZmOrphan94* with YFP^N^::*ZmbHLH80* or YFP^N^::*ZmbHLH90*. No signal was detected when ZmOrphan94 was co-transformed with the unrelated proteins Akin10 or Akin3, thus validating the observed interactions. We also observed that neither ZmCPP8 nor ZmHB87 interacts with either ZmbHLH80 or ZmbHLH90 (data not shown).

### ZmOrphan94 Acts as a Repressor and Impairs ZmbHLH90-Mediated *ZmPEPC1* Trans-Activation

**Figure 3:**
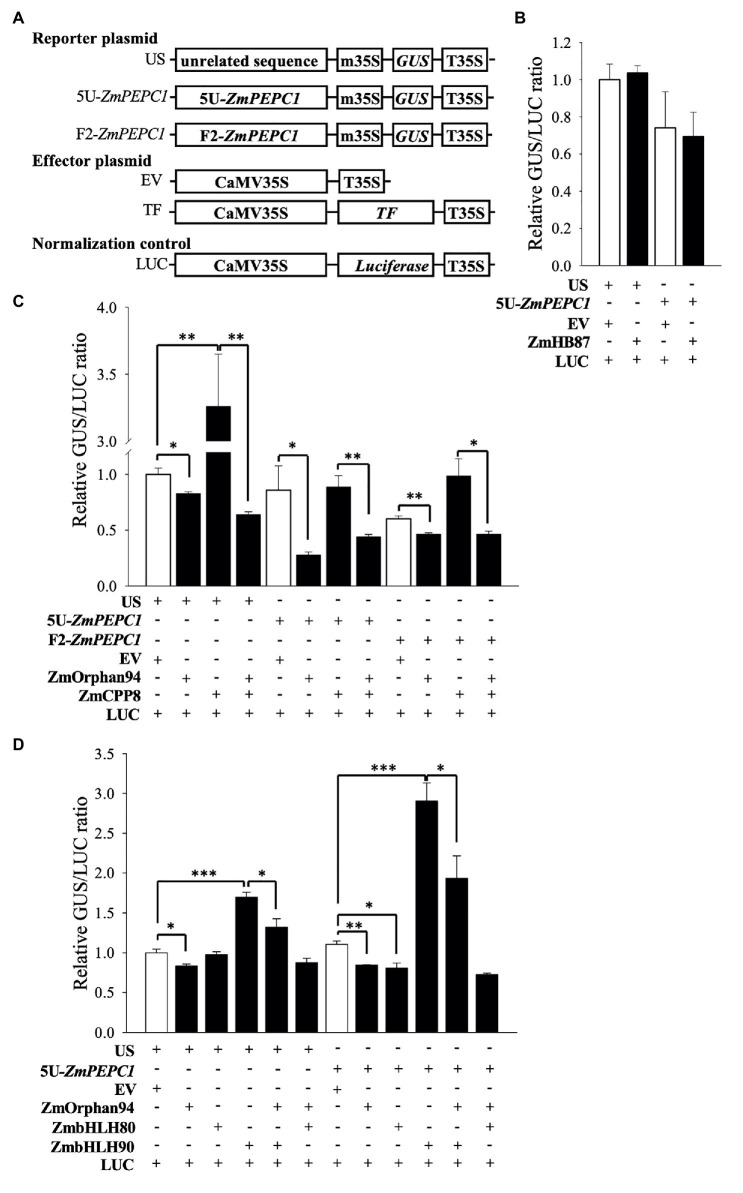
Trans-activation assays to test the activity of the novel TFs. **(A)** Schematic representation of the constructs used in trans-activation assays in maize mesophyll protoplasts. T35S, cauliflower mosaic virus 35S terminator; EV, empty vector; m35S, minimal cauliflower mosaic virus 35S promoter; CaMV35S, full cauliflower mosaic virus 35S promoter; GUS, *β-glucuronidase*; LUC, *luciferase*; US, reporter vector harboring an Unrelated Sequence; 5 U*-ZmPEPC1* and F2*-ZmPEPC1*, reporter vectors harboring the *ZmPEPC1* upstream region fragments (5 U and F2, respectively) used in Y1H screening. Trans-activation activity of ZmHB87 **(B)**, ZmCPP8 and ZmOrphan94 individually and co-transformed **(C)**, and ZmOrphan94 individually and co-transformed with ZmbHLH80 or ZmbHLH90 **(D)** represented as a GUS/LUC ratio. Data represent means ± SEM (*n* = 3). Differences are statistically significant (*t*-test, ^*^p < 0.05; ^**^p < 0.01; and ^***^p < 0.001).

To determine trans-activation activity of the novel TFs on the *ZmPEPC1* promoter, we conducted a trans-activation assay in maize M protoplasts (prepared from etiolated seedlings). We transiently transformed the protoplasts with reporter constructs (e.g., 5 U-*ZmPEPC1*::m35::GUS) and effector plasmids expressing TFs under the control of the CaMV35S promoter (Figure 3A). Co-transformation of maize protoplasts with the reporter US (containing an unrelated DNA sequence before the minimal 35S) together with either EV or ZmHB87 did not change the GUS/LUC ratio (Figure 3B). The same result was observed when the reporter 5 U-*ZmPEPC1* was co-transformed with either EV or ZmHB87, indicating that ZmHB87 has no trans-activation activity. Regarding ZmCPP8, it activated the US reporter vector, but its activation activity on the F2-*ZmPEPC1*, which contains the fragment bound by ZmCPP8, was not statistically significant (Figure 3C). These results suggest that ZmCPP8 may act as an activator and that the US reporter contains *cis*-element(s) recognized by ZmCPP8. According to our data, ZmOrphan94 acts as a transcriptional repressor. When ZmOrphan94 was co-transformed with either US, 5 U-*ZmPEPC1*, or F2-*ZmPEPC1* reporters, it always reduced the GUS/LUC ratio, as compared with the EV. In addition, the strongest repression was observed when ZmOrphan94 was co-transformed with 5 U-*ZmPEPC1* reporter (Figure 3C), which contains the *ZmPEPC1* upstream fragment where ZmOrphan94 binds (Figure 1B).

Our BIFC assays showed that ZmOrphan94 forms multiple heterodimers (Figure 2). It interacts with ZmCPP8, identified as binding to the F2-*ZmPEPC1* fragment, as well as ZmbHLH80 and ZmbHLH90, which were previously shown as binding to 5 U-*ZmPEPC1* (to which ZmOrphan94 also binds). To understand the role of these interactions regulating *ZmPEPC1* promoter activity, we also analyzed the trans-activation activity of ZmOrphan94 when acting together with the interacting TFs. Given that ZmCPP8 and ZmOrphan94 interact with each other but bind to different *ZmPEPC1* promoter fragments, we analyzed their trans-activation activity on both fragments. Consistent with its specific binding to the F2-*ZmPEPC1* fragment, ZmCPP8 did not show trans-activation activity on the 5 U-*ZmPEPC1* reporter vector, as compared with the EV (Figure 3C). However, when ZmCPP8 was co-transformed with ZmOrphan94, we observed a reduction of the GUS/LUC ratio for all the analyzed reporter vectors, as compared with that observed for ZmCPP8 alone (Figure 3C). When the ZmOrphan94 was co-transformed with the ZmbHLH80, we did not observe changes in the GUS/LUC ratio for US and 5 U-*ZmPEPC1*, as compared with those of ZmOrphan94 or ZmbHLH80 alone (Figure 3D). However, when ZmOrphan94 and ZmbHLH90 were co-transformed, ZmOrphan94 reduced the ZmbHLH90-mediated activation observed with US and 5 U-*ZmPEPC1* reporter vectors (Figure 3D), clearly indicating that ZmOrphan94 impairs the activation of *ZmPEPC1* caused by ZmbHLH90.

### *ZmOrphan94* and *ZmbHLH90* Show Similar Expression Profile

**Figure 4:**
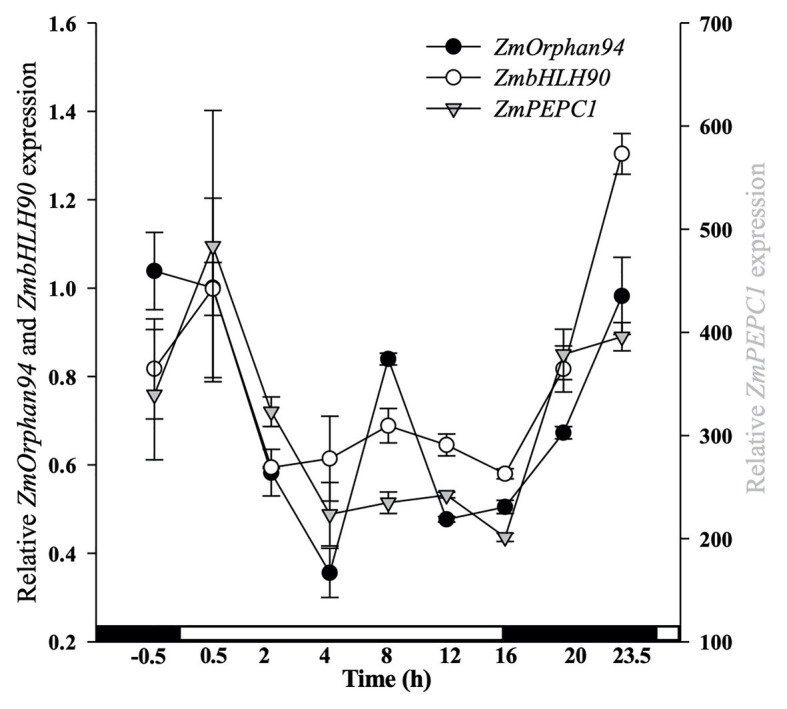
Analysis of diurnal gene expression for *ZmOrphan94*, *ZmbHLH90*, and *ZmPEPC1*. The transcript level was assessed by RT-qPCR over a 24 h period. On the *x*-axis, white and black boxes indicate light and dark periods, respectively. Transcript level values were normalized to the expression of *ZmActin1* (GRMZM2G126010). Data represent means ± SEM (*n* = 3).

To understand the role of the interaction between ZmOrphan94 and ZmbHLH90 regulating *ZmPEPC1* gene expression, we analyzed the gene expression pattern of *ZmOrphan94*, *ZmbHLH90*, and *ZmPEPC1* over a period of 24 h. As shown in Figure 4, *ZmOrphan94* and *ZmbHLH90* have a similar diurnal transcript profile. Moreover, despite the difference in the transcript levels observed between these TFs and their target gene *ZmPEPC1*, all three genes show a very similar diurnal expression pattern. The three genes have a peak of expression at the end of the night or at the beginning of the day and all three are downregulated during the 1st hour (0.5–4 h after dawn) of the day. After this, with the exception of *ZmOrphan94*, which shows an unexpected increase of expression in the middle of the photoperiod (8 h after dawn), the transcript level of these genes is downregulated till the end of the photoperiod (16 h). The expression of the three genes is then induced all night long to reach the peak at the end of the night (0.5 h pre-dawn)/beginning of the day (0.5 h after dawn; Figure 4). The high correlation between *ZmOrphan94*, *ZmbHLH90*, and *ZmPEPC1* gene expression patterns indicates that ZmOrphan94 and ZmbHLH90 may act together to regulate *ZmPEPC1* gene expression.

### ZmOrphan94 Binds to Different CACA Motifs Within the *ZmPEPC1* Upstream Region and One of Its Binding Sites Overlaps With the Binding Site of ZmbHLH90

**Figure 5:**
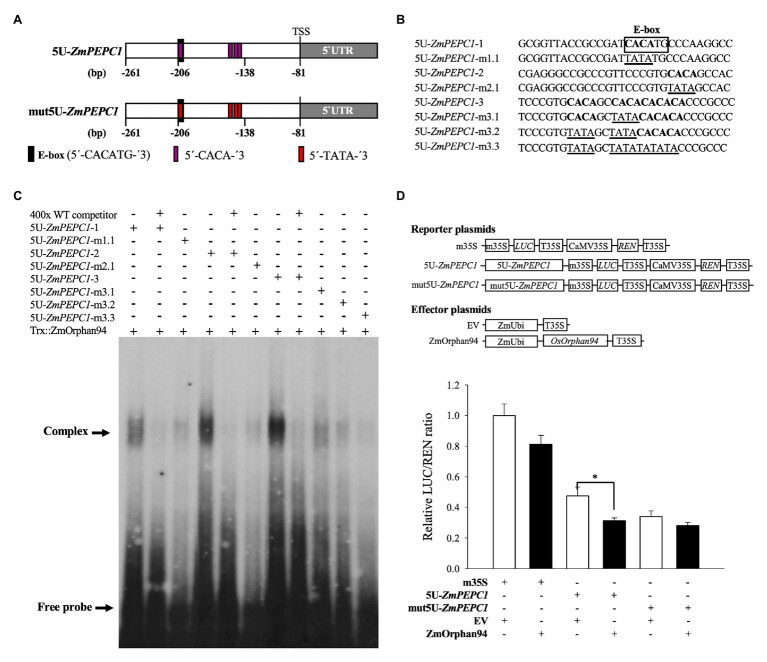
Analysis of the interactions between ZmOrphan94 and the *cis*-elements present in the 5 U *ZmPEPC1* fragment. **(A)** Schematic representation of the 5 U fragment (within *ZmPEPC1* upstream region) indicating the relative positions of the E-box (ZmbHLH90 binding site; black rectangle) and 5'-CACA-3' (ZmOrphan94 binding site; magenta rectangle) sequences, as well as its mutated form (mut5U-*ZmPEPC1*). mut5U-*ZmPEPC1* fragment has the ZmOrphan94 binding sites mutated from 5'-CACA-3' to 5'-TATA-3'. Nucleotide numbers refer to the *ZmPEPC1* translational start codon ATG (+1). **(B)** Nucleotide sequences of DNA probes used in electromobility shift assays (EMSA). Putative ZmOrphan94 binding sites (5'-CACA-3') are represented in bold. The CACA sequences mutated to TATA are underlined. The binding site for ZmbHLH90 (E-box) is indicated. **(C)** EMSA of the Trx::ZmOrphan94 with probes derived from the 5 U *ZmPEPC1* upstream region. The “+” and “−” indicate presence and absence of respective protein or probe. The “400x” indicates a 400-time excess of unlabeled wild-type (WT) probe. **(D)** Trans-activation assay to analyze the function of the ZmOrphan94 binding sites in the 5 U-*ZmPEPC1* fragment. Data represent means ± SEM (*n* = 10–12). Differences are statistically significant (*t*-test, ^*^p < 0.05).

Based on the trans-activation data, ZmOrphan94 impairs ZmbHLH90-mediated *ZmPEPC1* activation (Figure 3D). Given that ZmOrphan94 and ZmbHLH90 bind to the same 5 U-*ZmPEPC1* upstream fragment, it is possible that the effect observed on the *ZmPEPC1* expression is due to their binding to the same *cis*-element or to different *cis*-elements in close proximity. To understand where ZmOrphan94 binds within 5 U-*ZmPEPC1*, we first reviewed the literature to search for *cis*-elements described as binding sites for CCT domain-containing proteins, such as ZmOrphan94. It is reported that *Arabidopsis* TIMING OF CAB EXPRESSION 1 (TOC1), a CCT domain-containing TF, can bind to a 5′-CACA-3′ sequence (Gendron et al., 2012). *In silico* analysis of the 5 U-*ZmPEPC1* fragment, revealed five predicted CACA motifs within the 5 U-*ZmPEPC1* sequence, with one of them overlapping with the ZmbHLH90 binding site (E-box; Figures 5A,B). To determine whether ZmOrphan94 binds to the 5'-CACA-3' sequences, we produced a full-length ZmOrphan94 recombinant protein and performed EMSA. As shown in Figure 5C, Trx::ZmOrphan94 bound to all 5 U-*ZmPEPC1* fragments containing CACA motifs (5 U-*ZmPEPC1-*1, 5 U-*ZmPEPC1-*2, and 5 U-*ZmPEPC1-*3), causing an uplift of the radiolabeled probes. The strongest band intensity was observed for 5 U-*ZmPEPC1-*3 probe, which contains multiple 5'-CACA-3' elements (Figure 5C). Binding of Trx::ZmOrphan94 to the labeled wild-type (WT) probes could be efficiently out-competed by unlabeled WT probes, thus validating the TF-DNA binding (Figure 5C). As a negative control, the probe containing CACA sequence was incubated with Trx alone and no gel mobility shift was observed (Supplementary Figure S2). In addition, Trx::ZmOrphan94 did not bind to 5 U-*ZmPEPC1-*0 probe lacking 5'-CACA-3' sequence (Supplementary Figure S2). To test whether ZmOrphan94 binds specifically to the CACA sequence(s) within the 5 U-*ZmPEPC1* fragment, we also generated mutated probes. As observed in Figure 5C, the mutations within the 5'-CACA-3' sequence led to a strong decrease in the Trx::ZmOrphan94-DNA complex band intensities.

In order to test the biological function of the ZmOrphan94 binding sites, we have performed an additional trans-activation assay, using as reporter the firefly LUC gene driven by either 5 U-*ZmPEPC1* fragment or mut5U-*ZmPEPC1* (5 U-*ZmPEPC1* sequence in which all ZmOrphan94 binding sites were mutated; Figure 5A). We observed that, when the reporter gene is driven by 5 U-*ZmPEPC1* fragment, ZmOrphan94 represses its activity (Figure 5D). However, when all ZmOrphan94 binding sites are mutated in the 5 U-*ZmPEPC1* sequence, the ZmOrphan94 repression activity is impaired (Figure 5D). This shows that indeed ZmOrphan94 binds *in vivo* to the CACA elements present in the 5 U-*ZmPEPC1* sequence and that this binding is essential for its function as transcriptional repressor.

Altogether, our results showed that ZmOrphan94 binds specifically to multiple 5'-CACA-3 sequences within the 5 U-*ZmPEPC1* upstream region, being this binding crucial for its function as repressor. Furthermore, we showed that one of the ZmOrphan94 binding sites within the 5 U-*ZmPEPC1* overlaps with the DNA-binding site of ZmbHLH90. This suggests a possible competition of ZmbHLH90 and ZmOrphan94 to the same binding site within the *ZmPEPC1* upstream region. However, the fact that ZmOrphan94 also binds to motifs in close proximity and that ZmOrphan94 and ZmbHLH90 proteins can interact may also underlie the observed impairment of the ZmbHLH90-mediated *ZmPEPC1* activation by ZmOrphan94.

### ZmOrphan94 Rice Homologue Does Not Bind to the *ZmPEPC1* Upstream Region

In our previous studies, we have shown that the rice TF OsbHLH112 and its two maize homologues, ZmbHLH80 and ZmbHLH90, bind to the same *ZmPEPC1* upstream region (Górska et al., 2019). Given that C_4_ plants evolved from the C_3_, we proposed that these ZmbHLHs were co-opted during evolution of C_4_ photosynthesis. To investigate whether ZmOrphan94 was also recruited from C_3_ plants, we first searched for a ZmOrphan94 rice homologue and checked whether this homologue binds to the *ZmPEPC1* upstream region. To identify a rice homologue of ZmOrphan94, BLASTp search of the *O. sativa* genome with the ZmOrphan94 protein sequence was performed. This search identified rice OsOrphan65 (LOC_Os05g51690.1) as the best hit, showing 76.4% amino acid identity to the ZmOrphan94 protein sequence. OsOrphan65 full-length CDS was cloned into pDEST22 to be in fusion with the GAL4 activation domain (AD) and a direct Y1H assay was performed. The 5 U-*ZmPEPC1* bait was transformed with OsOrphan65, EV, and ZmbHLH90 (positive control) and the growth of the transformed bait strain was analyzed on a CM/-Trp/-His selection medium supplemented with increasing concentrations of 3-AT. Our results showed that OsOrphan65 does not interact with the 5 U-*ZmPEPC1* fragment. The growth of the 5 U-*ZmPEPC1* bait strain transformed with OsOrphan65 or EV was repressed on a CM/-Trp/-His selection media supplemented with 5 mM 3-AT (Supplementary Figure S3). On the other hand, the 5 U-*ZmPEPC1* bait strain transformed with ZmbHLH90 (positive control) grew on CM/-Trp/-His + 20 mM 3-AT (Supplementary Figure S3).

### *ZmOrphan94* Is Preferentially Expressed in Maize BS Cells

**Figure 6:**
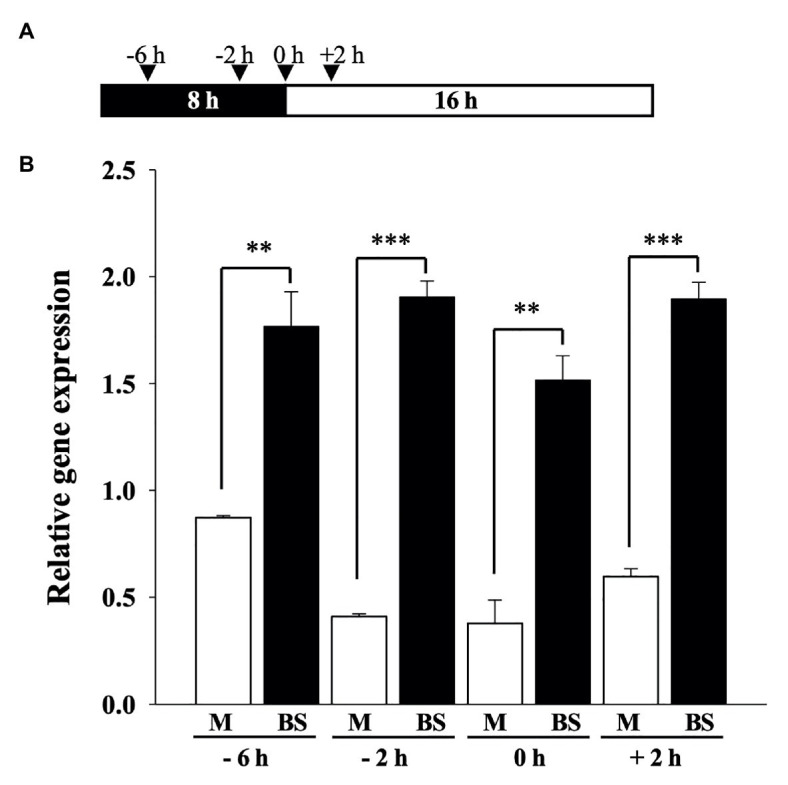
Analysis of *ZmOrphan94* transcript level in mesophyll (M) and bundle sheath (BS) cells. **(A)** Schematic representation of the sampling time points. Black and white box indicate dark and light period, respectively. Arrowheads indicate sampling points. **(B)** *ZmOrphan94* expression in BS and M cells in the analyzed time points. *ZmOrphan94* transcript levels were analyzed by RT-qPCR and normalized against the expression of two housekeeping genes (GRMZM2G144843 and GRMZM2G044552). Data represent means ± SEM (*n* = 3). Statistical significance (*t*-test, ^**^p < 0.01 and ^***^p < 0.001).

M-specific expression of *ZmPEPC1* is known to be regulated at transcriptional level, activated in M, and repressed in BS cells (Kausch et al., 2001). Our results indicate that ZmOrphan94 is a *ZmPEPC1* transcriptional repressor that binds to the *ZmPEPC1* upstream region involved in the cell-specific *ZmPEPC1* gene expression. To better understand the role of ZmOrphan94 in the regulation of *ZmPEPC1* gene expression, we analyzed *ZmOrphan94* transcript levels in M and BS cells. For this, we purified M and BS cells from fully expanded maize third leaves at several time points, in the dark (−6 and −2 h), at the transition between light and dark (0 h) and during the light (+2 h, Figure 6A). Purity of isolated M and BS cells was assessed using the cell‐specific gene markers, *ZmPEPC1* for M and *ZmNADP-ME* for BS cells (Supplementary Figure S4). As shown in Figure 6B, *ZmOrphan94* shows a much higher transcript level in BS than in M cells for all the time points analyzed. The higher expression of *ZmOrphan94* in BS cells, as compared with M cells, is particularly striking 2 h before dawn, and at dawn, when *ZmOrphan94* transcript level is, respectively, 4.75 and 3.9 times higher in BS than in M cells. The smallest difference in *ZmOrphan94* transcript accumulation between the two cell types is observed at 6 h before dawn. Nevertheless, at this time point, *ZmOrphan94* is approximately two times more expressed in BS than in M cells (Figure 6B).

## Discussion

### The Role of ZmHB87 and ZmCPP8 in the Regulation of *ZmPEPC1* Gene Expression

In this study, we have identified three novel TFs binding to the *ZmPEPC1* upstream region (ZmOrphan94, ZmHB87, and ZmCPP8). However, despite our efforts, the role of ZmHB87 and ZmCPP8 on the regulation of *ZmPEPC1* gene expression is still not clear and requires further studies. ZmHB87 is a member of the homeobox TF family reported to form homo- and heter-odimers (Meijer et al., 2000; Mukherjee and Brocchieri, 2010). Nevertheless, under our experimental conditions, ZmHB87 did not form homodimers, did not interact with the other TFs analyzed, and did not show any trans-activation activity on the *ZmPEPC1* upstream region. In addition, *in silico* analysis of ZmHB87 amino acid sequence using TargetP (Emanuelsson et al., 2000) revealed that besides the NLS, ZmHB87 also carries a chloroplast transit peptide (cTP) localized at N-terminus of the protein (Supplementary Figure S1B). Overall, our results suggest that ZmHB87 may not regulate *ZmPEPC1* gene expression.

ZmCPP8 is a member of the cysteine-rich polycomb-like protein (CPP) TF family. Members of this family contain a highly conserved cysteine rich domain (CXC) within their DNA binding motif (Hauser et al., 2000; Schmit et al., 2009). CPPs are known to be involved in different processes, such as female and male sterility in *Arabidopsis* (Andersen et al., 2007), salt tolerance in rice (Almeida et al., 2016), and root nodule formation in soybean (Cvitanich et al., 2000). According to our results, ZmCPP8 may also be involved in the *ZmPEPC1* regulation in maize. ZmCPP8 was identified by Y1H as binding to the *ZmPEPC1* upstream region. Nevertheless, the addition of 5 mM 3-AT was enough to impair the ZmCPP8 binding to the F2-*ZmPEPC1* fragment, suggesting a weak interaction. In addition, the trans-activation assays to test ZmCPP8 activity showed a stronger activation of the US reporter (control) as compared to the F2-*ZmPEPC1* reporter, to which ZmCPP8 binds specifically. Thus, to determine whether ZmCPP8 is indeed involved in the *ZmPEPC1* regulation, further work is needed. First, it is essential to identify the *cis*-regulatory elements within the *ZmPEPC1* upstream region where ZmCPP8 can bind. LIN54 is perhaps the best characterized member of the CPP family and it was shown as binding to the *cis*-elements CDE (5'-TAGCGCGGT-3') and CHR (5'-TTYRAA-3', where Y is a pyrimidine and R is a purine; Schmit et al., 2009; Marceau et al., 2016). *In silico* analysis of the F2-*ZmPEPC1* sequence revealed the presence of CDE and CHR resembling motifs (Supplementary Table S1). Therefore, the next step would be to determine whether ZmCPP8 binds to these elements *in vitro* and *in vivo*. Recently, members of the maize CPP family have been characterized in terms of their response to abiotic stresses (Song et al., 2016). *ZmCPP8* (named *ZmCPP11* in that study) was upregulated in response to cold and heat treatment. The expression of *ZmCPP8* was strongly induced after 12 h of cold (4°C) and heat (42°C) treatments (Song et al., 2016). These findings suggest a putative role of ZmCPP8 in response to cold and heat stress in maize. Given that *PEPC1* transcript level is upregulated under cold stress (Li et al., 2019), it would be interesting to determine whether ZmCPP8 mediates the effect of cold on *ZmPEPC1* gene expression.

### ZmOrphan94 May Play an Important Role in *ZmPEPC1* Regulatory Network

Our data indicate that ZmOrphan94 forms multiple heterodimers with other TFs binding to the *ZmPEPC1* upstream region. Interactions of ZmOrphan94 with other proteins occur likely through the CCT domain shown to mediate protein-protein interactions (Kurup et al., 2000; Wenkel et al., 2006). ZmOrphan94 forms homodimers, suggesting intra-family interactions, but also interacts with TFs from other TF families (e.g., CPP and bHLH). Even though intra-family interactions are most common and have been extensively studied (Amoutzias et al., 2008), the importance of cross-family interactions in a global regulatory network is well known (Bemer et al., 2017). The ability to interact with various TFs belonging to different TF families suggests that ZmOrphan94 plays an important role in the transcriptional network regulating *ZmPEPC1* gene expression, thus increasing its complexity and flexibility. There are several possible ways on how TF dimerization may affect TF activity. First, a TF complex may increase or reduce binding affinity of individual TFs to DNA. For example, in *Arabidopsis thaliana* a complex of auxin-response factor (ARF6) with PIF4/BZR1 increased DNA-binding affinity to ARF6/PIF4/BZR1 common targets, whereas decreased affinity for ARF6 specific targets (Oh et al., 2014). The effect of heterodimer formation on DNA binding specificity was also reported for MADS-domain containing proteins. The distinct binding preferences of MADS-box SEPALLATA3 (SEP3) and AGAMOUS (AG) TF heterodimer are involved in specification of reproductive organs in *Arabidopsis* (Smaczniak et al., 2017). TF-TF interactions may also affect the trans-activation activity of the individual TFs. For example, in sweet potato, bHLH heterodimerization is involved in plant defense against herbivory (Chen et al., 2016). In response to wounding, the TF IbbHLH3 activates the defense network through binding to the promoter and activation of IbNAC1 gene expression (Chen et al., 2016). To terminate the response, the IbbHLH3-IbbHLH4 heterodimer, which downregulates IbNAC1 expression, competes with IbbHLH3 homodimer for binding to the IbNAC1 promoter (Chen et al., 2016). Different transcriptional activity of TF-TF heterodimer, comparing to individual TFs, was also reported for the TFs involved in plant response to water deficit and osmotic stress conditions. ANAC096, a NAC (for NAM, ATAF1/2, and CUC2) TF, and ABF2, a bZIP-type TF, interact and are activators of *RD29A* gene expression. When co-transformed, ANAC096 and ABF2 act synergistically to activate *RD29A* expression (Xu et al., 2013). According to our results, ZmOrphan94 acts as a transcriptional repressor and co-expression of ZmOrphan94 with the identified activators, ZmCPP8 and ZmbHLH90, reduces the transactivation activity of the individual TFs on the *ZmPEPC1* promoter. Nevertheless, our transactivation experimental setup did not allow to determine whether this effect is due to competition for the same *cis*-element(s) or to heterodimerization, and its effect on DNA-binding or on TF trans-activation activity. Therefore, to understand in more detail the effect of ZmOrphan94 on the trans-activation activity of ZmCPP8 and ZmbHLH90, further work is needed.

### ZmOrphan94 Is Part of a Regulatory Mechanism That Downregulates *ZmPEPC1* Gene Expression in Bundle Sheath Cells

**Figure 7:**
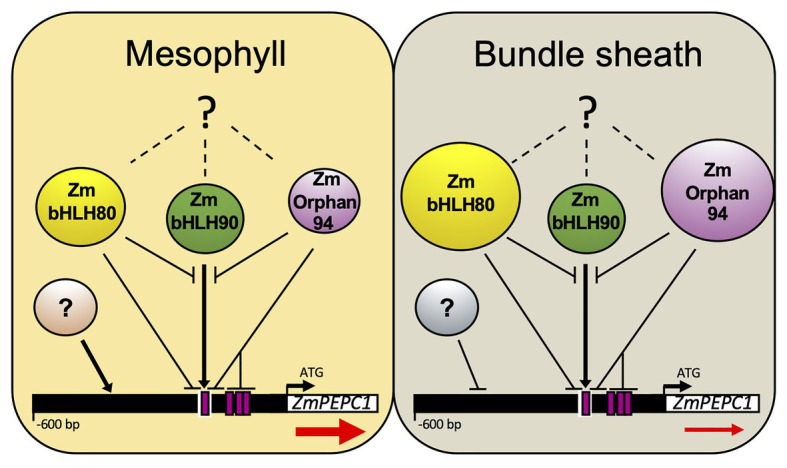
Model integrating the contribution of ZmOrphan94, ZmbHLH80, and ZmbHLH90 to regulate M-specific *ZmPEPC1* expression. ZmOrphan94 and ZmbHLH80 are repressors, whereas ZmbHLH90 is an activator of *ZmPEPC1* expression. ZmOrphan94 and ZmbHLH80 impair ZmbHLH90-mediated *ZmPEPC1* activation through competitive binding to the same *cis*-element (E-Box; white rectangle) and/or heterodimerization. ZmOrphan94 can also impair ZmbHLH90-mediated *ZmPEPC1* activation through its binding to the CACA motifs (magenta rectangles) present within the *ZmPEPC1* upstream region, being that one of them is present within the E-Box (white rectangle). *ZmOrphan94* and *ZmbHLH80* are preferentially expressed in BS cells, thus having a predominant role repressing *ZmPEPC1* expression in this cell type. Dashed lines with question marks represent mechanisms that may regulate ZmOrphan94, ZmbHLH80, and ZmbHLH90 post-transcriptionally. Question marks in circles represent yet unidentified TFs that may be involved in M-specific *ZmPEPC1* expression. Arrows and horizontal lines indicate activation and repression, respectively. Sizes of ZmOrphan94, ZmbHLH80, and ZmbHLH90 circles represent differences in transcript abundance. Red arrows below *ZmPEPC1* gene indicate the difference in *ZmPEPC1* transcript abundance between M and BS cells.

Our results clearly show that ZmOrphan94 is part of the transcriptional network regulating *ZmPEPC1* gene expression. Based on our findings, we propose that together with the previously identified TFs, ZmbHLH80 and ZmbHLH90, ZmOrphan94 contributes to the M-specific *ZmPEPC1* gene expression (Figure 7). According to our results, ZmOrphan94 regulates *ZmPEPC1* transcript level in a similar manner as ZmbHLH80 (Górska et al., 2019). ZmOrphan94 acts as a repressor and impairs ZmbHLH90-mediated *ZmPEPC1* activation. This impairment is clear but may occur due to different reasons: (a) ZmOrphan94 and ZmbHLH90 form heterodimers, thus impairing ZmbHLH90 binding and/or ZmbHLH90 trans-activation activity; (b) ZmOrphan94 and ZmbHLH90 compete to the same *cis*-element; (c) ZmOrphan94 and ZmbHLH90 bind to *cis*-elements in close proximity and their interaction weakens ZmbHLH90 trans-activation activity; and (d) ZmOrphan94 has a repressor activity that overcomes ZmbHLH90 activator activity. Given that *ZmOrphan94* shows higher transcript levels in BS cells, as compared to M cells, we propose that together with BS-preferentially expressed ZmbHLH80, ZmOrphan94 plays a role in maintaining the *ZmPEPC*1 transcript levels low in BS cells (Figure 7). In maize, cell-specific downregulation has been reported for the *RbcS* gene family encoding RuBisCO small subunit. *RbcS* genes become BS-specific upon illumination (Sheen and Bogorad, 1986, 1987) and sequences within the upstream region and 3'UTR fragment of *RbcS-m3* have been involved in this expression pattern (Viret et al., 1994). Moreover, it was proposed that a TF belonging to the Krüppel-type zinc finger family, TRANSCRIPTION REPRESSOR MAIZE 1 (ZmTTM1), may be involved in this regulation. Though *ZmTTM1* expression, unlike *ZmOrphan94*, is not more abundant in a given cell type, mutations of any of its binding sites within *RbcS-m3* gene eliminate the repression of the *RBCS-m3* reporter gene in M cells (Xu et al., 2001). Interestingly, ZmOrphan94, ZmbHLH80, and ZmbHLH90 bind to the *ZmPEPC1* upstream region, within the conserved nucleotide sequences (CNSs) motifs recently identified by Gupta et al. (2020). ZmOrphan94 binds within CNS-1 and CNS-3B motifs, whereas ZmbHLH80 and ZmbHLH90 bind to the CNS-1. The CNS motifs are conserved among the C_4_ *PEPC* genes from the Panicoid clade and are essential for driving M-cell specific gene expression in rice (Gupta et al., 2020), highlighting the importance of these CNS motifs as well as their binding TFs for the C_4_ *PEPC* cell-specific gene expression. The current attempts to engineer the C_4_ metabolism into rice (C_3_ plant) require cell-specific accumulation of C_4_ enzymes. To successfully accomplish this ambitious goal, we need to identify and characterize the function of the different *cis*-regulatory elements as well as the binding TFs and the molecular mechanisms underlying this feature.

Taken together, our data reveal the importance of the regulatory mechanisms within BS cells that contribute to the M-specific *ZmPEPC1* gene expression. We show that at least two TFs, ZmOrphan94 and ZmbHLH80, act to suppress *ZmPEPC1* gene expression in BS cells. It is likely that ZmbHLH80 was co-opted from the ancestral C_3_ pathway, whereas ZmOrphan94 was recruited during evolution of C_4_ photosynthesis.

## Data Availability Statement

The raw data supporting the conclusions of this article will be made available by the authors, without undue reservation.

## Author Contributions

AG and PG conceived the project, performed the experiments, analyzed the data, and wrote the manuscript. AB, AZ, TS, PC, and TL performed the experiments and analyzed the data. MO conceived the project and revised the manuscript. CP conceived the project and raised funds. NS conceived the project, raised funds, analyzed the data, and revised the manuscript. All authors contributed to the article and approved the submitted version.

### Conflict of Interest

The authors declare that the research was conducted in the absence of any commercial or financial relationships that could be construed as a potential conflict of interest.

## References

[ref1] Akyildiz M. Gowik U. Engelmann S. Koczor M. Streubel M. Westhoff P. (2007). Evolution and function of a cis-regulatory module for mesophyll-specific gene expression in the C4 dicot *Flaveria trinervia*. Plant Cell 19, 3391–3402. 10.1105/tpc.107.053322, PMID: 17993624 PMC2174892

[ref2] Almeida D. M. Gregorio G. B. Oliveira M. M. Saibo N. J. M. (2016). Five novel transcription factors as potential regulators of *OsNHX1* gene expression in a salt tolerant rice genotype. Plant Mol. Biol. 93, 61–77. 10.1007/s11103-016-0547-7, PMID: 27766460

[ref3] Amoutzias G. D. Robertson D. L. Van de Peer Y. Oliver S. G. (2008). Choose your partners: dimerization in eukaryotic transcription factors. Trends Biochem. Sci. 33, 220–229. 10.1016/j.tibs.2008.02.002, PMID: 18406148

[ref4] Andersen S. U. Algreen-Petersen R. G. Hoedl M. Jurkiewicz A. Cvitanich C. Braunschweig U.. (2007). The conserved cysteine-rich domain of a tesmin/TSO1-like protein binds zinc in vitro and TSO1 is required for both male and female fertility in *Arabidopsis thaliana*. J. Exp. Bot. 58, 3657–3670. 10.1093/jxb/erm215, PMID: 18057042

[ref5] Aubry S. Brown N. J. Hibberd J. M. (2011). The role of proteins in C3 plants prior to their recruitment into the C4 pathway. J. Exp. Bot. 62, 3049–3059. 10.1093/jxb/err012, PMID: 21321052

[ref500] Bauwe H. Hagemann M. Fernie A. R. (2010). Photorespiration: players, partners and origin. Trends Plant Sci. 15, 330–336. PMID: 20403720 10.1016/j.tplants.2010.03.006

[ref6] Bemer M. van Dijk A. D. J. Immink R. G. H. Angenent G. C. (2017). Cross-family transcription factor interactions: an additional layer of gene regulation. Trends Plant Sci. 22, 66–80. 10.1016/j.tplants.2016.10.007, PMID: 27814969

[ref7] Borba A. R. Serra T. S. Górska A. Gouveia P. Cordeiro A. M. Reyna-Llorens I.. (2018). Synergistic binding of bHLH transcription factors to the promoter of the maize NADP-ME gene used in C4 photosynthesis is based on an ancient code found in the ancestral C3 state. Mol. Biol. Evol. 35, 1690–1705. 10.1093/molbev/msy060, PMID: 29659975 PMC5995220

[ref8] Brown N. J. Newell C. A. Stanley S. Chen J. E. Perrin A. J. Kajala K.. (2011). Independent and parallel recruitment of preexisting mechanisms underlying C₄ photosynthesis. Science 331, 1436–1439. 10.1126/science.1201248, PMID: 21415351

[ref9] Cavalar M. Phlippen Y. Kreuzaler F. Peterhänsel C. (2007). A drastic reduction in DOF1 transcript levels does not affect C4-specific gene expression in maize. J. Plant Physiol. 164, 1665–1674. 10.1016/j.jplph.2006.09.008, PMID: 17178169

[ref10] Chen S. P. Kuo C. H. Lu H. H. Lo H. S. Yeh K. W. (2016). The sweet potato NAC-domain transcription factor IbNAC1 is dynamically coordinated by the activator IbbHLH3 and the repressor IbbHLH4 to reprogram the defense mechanism against wounding. PLoS Genet. 12:e1006397. 10.1371/journal.pgen.1006397, PMID: 27780204 PMC5079590

[ref11] Cordeiro A. M. Figueiredo D. D. Tepperman J. Borba A. R. Lourenço T. Abreu I. A.. (2016). Rice phytochrome-interacting factor protein OsPIF14 represses *OsDREB1B* gene expression through an extended N-box and interacts preferentially with the active form of phytochrome B. Biochim. Biophys. Acta, Gene Regul. Mech. 1859, 393–404. 10.1016/j.bbagrm.2015.12.008, PMID: 26732823 PMC4824199

[ref12] Covshoff S. Furbank R. T. Leegood R. C. Hibberd J. M. (2013). Leaf rolling allows quantification of mRNA abundance in mesophyll cells of sorghum. J. Exp. Bot. 64, 807–813. 10.1093/jxb/ers286, PMID: 23077203

[ref13] Cvitanich C. Pallisgaard N. Nielsen K. A. Hansen A. C. Larsen K. Pihakaski-Maunsbach K.. (2000). CPP1, a DNA-binding protein involved in the expression of a soybean leghemoglobin C3 gene. Proc. Natl. Acad. Sci. U. S. A. 97, 8163–8168. 10.1073/pnas.090468497, PMID: 10859345 PMC16687

[ref14] Emanuelsson O. Nielsen H. Brunak S. von Heijne G. (2000). Predicting subcellular localization of proteins based on their N-terminal amino acid sequence. J. Mol. Biol. 300, 1005–1016. 10.1006/jmbi.2000.3903, PMID: 10891285

[ref15] Figueiredo D. D. Barros P. Cordeiro A. M. Serra T. Lourenço T. Subhash C.. (2012). Seven zinc-finger transcription factors are novel regulators of the stress responsive gene *OsDREB1B*. J. Exp. Bot. 63, 695–709. 10.1093/jxb/ers035, PMID: 22412187

[ref16] Furbank R. T. Taylor W. C. (1995). Regulation of photosynthesis in C3 and C4 plants: a molecular approach. Plant Cell 7, 797–807. 10.2307/3870037, PMID: 12242386 PMC160868

[ref17] Gendron J. M. Pruneda-Paz J. L. Doherty C. J. Gross A. M. Kang S. E. Kay S. A. (2012). *Arabidopsis* circadian clock protein, TOC1, is a DNA-binding transcription factor. Proc. Natl. Acad. Sci. 109, 3167–3172. 10.1073/pnas.1200355109, PMID: 22315425 PMC3286946

[ref18] Górska A. M. Gouveia P. Borba A. R. Zimmermann A. Serra T. S. Lourenco T. F.. (2019). ZmbHLH80 and ZmbHLH90 transcription factors act antagonistically and contribute to regulate *PEPC1* cell-specific gene expression in maize. Plant J. 99, 270–285. 10.1111/tpj.14323, PMID: 30900785

[ref19] Gowik U. Burscheidt J. Akyildiz M. Schlue U. Koczor M. Streubel M.. (2004). Cis-regulatory elements for mesophyll-specific gene expression in the C4 plant *Flaveria trinervia*, the promoter of the C4 phosphoenolpyruvate carboxylase gene. Plant Cell 16, 1077–1090. 10.1105/tpc.019729, PMID: 15100398 PMC423201

[ref20] Gowik U. Schulze S. Saladié M. Rolland V. Tanz S. K. Westhoff P.. (2016). A MEM1-like motif directs mesophyll cell-specific expression of the gene encoding the C4 carbonic anhydrase in Flaveria. J. Exp. Bot. 68, 311–320. 10.1093/jxb/erw475, PMID: 28040798 PMC5853542

[ref21] Gupta S. D. Levey M. Schulze S. Karki S. Emmerling J. Streubel M.. (2020). The C4Ppc promoters of many C4 grass species share a common regulatory mechanism for gene expression in the mesophyll cell. Plant J. 101, 204–216. 10.1111/tpj.14532, PMID: 31529521

[ref22] Hauser B. A. He J. Q. Park S. O. Gasser C. S. (2000). TSO1 is a novel protein that modulates cytokinesis and cell expansion in *Arabidopsis*. Development 127, 2219–2226. PMID: 10769245 10.1242/dev.127.10.2219

[ref23] Hellens R. P. Allan A. C. Friel E. N. Bolitho K. Grafton K. Templeton M. D.. (2005). Transient expression vectors for functional genomics, quantification of promoter activity and RNA silencing in plants. Plant Methods 1:13. 10.1186/1746-4811-1-13, PMID: 16359558 PMC1334188

[ref24] Hibberd J. M. Covshoff S. (2010). The regulation of gene expression required for C4 photosynthesis. Annu. Rev. Plant Biol. 61, 181–207. 10.1146/annurev-arplant-042809-112238, PMID: 20192753

[ref25] Kajala K. Brown N. J. Williams B. P. Borrill P. Taylor L. E. Hibberd J. M. (2012). Multiple *Arabidopsis* genes primed for recruitment into C4 photosynthesis. Plant J. 69, 47–56. 10.1111/j.1365-313X.2011.04769.x, PMID: 21883556

[ref26] Kano-Murakami Y. Suzuki I. Sugiyama T. Matsuoka M. (1991). Sequence-specific interactions of a maize factor with a GC-rich repeat in the phosphoenolpyruvate carboxylase gene. Mol. Gen. Genet. 225, 203–208. 10.1007/BF00269849, PMID: 2005862

[ref27] Kausch A. P. Owen T. P. Zachwieja S. J. Flynn A. R. Sheen J. (2001). Mesophyll-specific, light and metabolic regulation of the C4 *PPCZm1* promoter in transgenic maize. Plant Mol. Biol. 45, 1–15. 10.1023/A:1006487326533, PMID: 11247600

[ref29] Kurup S. Jones H. D. Holdsworth M. J. (2000). Interactions of the developmental regulator ABI3 with proteins identified from developing *Arabidopsis* seeds. Plant J. 21, 143–155. 10.1046/j.1365-313x.2000.00663.x, PMID: 10743655

[ref30] Li M. Sui N. Lin L. Yang Z. Zhang Y. (2019). Transcript profiling revealed genes involved in response to cold stress in maize. Funct. Plant Biol. 46, 830–844. 10.1071/FP19065, PMID: 31217070

[ref31] Marceau A. H. Felthousen J. G. Goetsch P. D. Iness A. N. Lee H. W. Tripathi S. M.. (2016). Structural basis for LIN54 recognition of CHR elements in cell cycle-regulated promoters. Nat. Commun. 7, 1–11. 10.1038/ncomms12301, PMID: 27465258 PMC4974476

[ref32] Markelz N. H. Costich D. E. Brutnell T. P. (2003). Photomorphogenic responses in maize seedling development. Plant Physiol. 133, 1578–1591. 10.1104/pp.103.029694, PMID: 14645729 PMC300715

[ref33] Matsuoka M. Kyozuka J. Shimamoto K. Kano-Murakami Y. (1994). The promoters of two carboxylases in a C4 plant (maize) direct cell-specific, light-regulated expression in a C3 plant (rice). Plant J. 6, 311–319. 10.1046/j.1365-313X.1994.06030311.x, PMID: 7920719

[ref34] Meijer A. H. de Kam R. J. D’Erfurth I. Shen W. Hoge J. H. C. (2000). HD-zip proteins of families I and II from rice: interactions and functional properties. Mol. Gen. Genet. 263, 12–21. 10.1007/PL00008671, PMID: 10732669

[ref35] Mukherjee K. Brocchieri L. (2010). Evolution of plant homeobox genes. eLS 2, 31–45. 10.1002/9780470015902.a0022865, PMID: 19734295 PMC2775110

[ref36] Nomura M. Sentoku N. Nishimura A. Lin J. H. Honda C. Taniguchi M.. (2000). The evolution of C4 plants: acquisition of cis-regulatory sequences in the promoter of C4-type pyruvate, orthophosphate dikinase gene. Plant J. 22, 211–221. 10.1046/j.1365-313x.2000.00726.x, PMID: 10849339

[ref37] Oh E. Zhu J. Y. Bai M. Y. Arenhart R. A. Sun Y. Wang Z. Y. (2014). Cell elongation is regulated through a central circuit of interacting transcription factors in the *Arabidopsis* hypocotyl. eLife 3, 1–19. 10.7554/eLife.03031, PMID: 24867218 PMC4075450

[ref38] Ouwerkerk P. B. Meijer A. H. (2001). Yeast one-hybrid screening for DNA-protein interactions. Curr. Protoc. Mol. Biol. 55, 12.12.1–12.12.12. 10.1002/0471142727.mb1212s55, PMID: 18265084

[ref39] Patel M. Siegel A. J. Berry J. O. (2006). Untranslated regions of FbRbcS1 mRNA mediate bundle sheath cell-specific gene expression in leaves of a C4 plant. J. Biol. Chem. 281, 25485–25491. 10.1074/jbc.M604162200, PMID: 16803877

[ref40] Portis A. R. Parry M. A. J. (2007). Discoveries in Rubisco (Ribulose 1,5-bisphosphate carboxylase/oxygenase): a historical perspective. Photosynth. Res. 94, 121–143. 10.1007/s11120-007-9225-6, PMID: 17665149

[ref41] Reeves G. Grangé-Guermente M. J. Hibberd J. M. (2017). Regulatory gateways for cell-specific gene expression in C4 leaves with Kranz anatomy. J. Exp. Bot. 68, 107–116. 10.1093/jxb/erw438, PMID: 27940469

[ref42] Reyna-Llorens I. Burgess S. J. Reeves G. Singh P. Stevenson S. R. Williams B. P.. (2018). Ancient duons may underpin spatial patterning of gene expression in C4 leaves. Proc. Natl. Acad. Sci. 115, 1931–1936. 10.1073/pnas.1720576115, PMID: 29432183 PMC5828626

[ref44] Sage R. F. (2004). The evolution of C4 photosynthesis. New Phytol. 161, 341–370. 10.1111/j.1469-8137.2004.00974.x 33873498

[ref45] Schmit F. Cremer S. Gaubatz S. (2009). LIN54 is an essential core subunit of the DREAM/LINC complex that binds to the *cdc2* promoter in a sequence-specific manner. FEBS J. 276, 5703–5716. 10.1111/j.1742-4658.2009.07261.x, PMID: 19725879

[ref46] Serra T. S. Figueiredo D. D. Cordeiro A. M. Almeida D. M. Lourenço T. Abreu I. A.. (2013). OsRMC, a negative regulator of salt stress response in rice, is regulated by two AP2/ERF transcription factors. Plant Mol. Biol. 82, 439–455. 10.1007/s11103-013-0073-9, PMID: 23703395

[ref47] Sheen J. (1991). Molecular mechanisms underlying the differential expression of maize pyruvate, orthophosphate dikinase genes. Plant Cell 3, 225–245. 10.1105/tpc.3.3.225, PMID: 1668653 PMC159995

[ref48] Sheen J. -Y. Bogorad L. (1986). Expression of the ribulose-1, 5-bisphosphate carboxylase large subunit gene and three small subunit genes in two cell types of maize leaves. EMBO J. 5:3417. 10.1002/j.1460-2075.1986.tb04663.x, PMID: 16453739 PMC1167374

[ref49] Sheen J. Y. Bogorad L. (1987). Differential expression of C4 pathway genes in mesophyll and bundle sheath cells of greening maize leaves. J. Biol. Chem. 262, 11726–11730. 10.1016/S0021-9258(18)60871-3, PMID: 2442151

[ref50] Smaczniak C. Muiño J. M. Chen D. Angenent G. C. Kaufmann K. (2017). Differences in DNA-binding specificity of floral homeotic protein complexes predict organ-specific target genes. Plant Cell 29, 1822–1835. 10.1105/tpc.17.00145, PMID: 28733422 PMC5590503

[ref51] Song X. Y. Zhang Y. Y. Wu F. C. Zhang L. (2016). Genome-wide analysis of the maize (*Zea may* L.) CPP-like gene family and expression profiling under abiotic stress. Genet. Mol. Res. 15, 1–11. 10.4238/gmr.15038023, PMID: 27525875

[ref52] Stockhaus J. Schlue U. Koczor M. Chitty J. A. Taylor W. C. Westhoff P. (1997). The promoter of the gene encoding the C4 form of phosphoenolpyruvate carboxylase directs mesophyll-specific expression in transgenic C4 Flaveria spp. Plant Cell 9, 479–489. 10.1105/tpc.9.4.479, PMID: 12237362 PMC156933

[ref53] Taniguchi M. Izawa K. Ku M. S. B. Lin J. -H. Saito H. Ishida Y.. (2000). Binding of cell type-specific nuclear proteins to the 5'-flanking region of maize C4 phosphoenolpyruvate carboxylase gene confers its differential transcription in mesophyll cells. Plant Mol. Biol. 44, 543–557. 10.1023/A:1026565027772, PMID: 11197328

[ref54] Viret J. F. Mabrouk Y. Bogorad L. (1994). Transcriptional photoregulation of cell-type-preferred expression of maize Rbcs-m3-3' and 5' sequences are involved. Proc. Natl. Acad. Sci. U. S. A. 91, 8577–8581. 10.1073/pnas.91.18.8577, PMID: 8078926 PMC44649

[ref55] Wenkel S. Turck F. Singer K. Gissot L. Le Gourrierec J. Samach A.. (2006). CONSTANS and the CCAAT box binding complex share a functionally important domain and interact to regulate flowering of *Arabidopsis*. Plant Cell 18, 2971–2984. 10.1105/tpc.106.043299, PMID: 17138697 PMC1693937

[ref56] Williams B. P. Burgess S. J. Reyna-Llorens I. Knerova J. Aubry S. Stanley S.. (2016). An untranslated cis-element regulates the accumulation of multiple C4 enzymes in *Gynandropsis gynandra* mesophyll cells. Plant Cell 28, 454–465. 10.1105/tpc.15.00570, PMID: 26772995 PMC4790868

[ref57] Wiludda C. Schulze S. Gowik U. Engelmann S. Koczor M. Streubel M.. (2012). Regulation of the photorespiratory GLDPA gene in C4 Flaveria: an intricate interplay of transcriptional and posttranscriptional processes. Plant Cell 24, 137–151. 10.1105/tpc.111.093872, PMID: 22294620 PMC3289567

[ref58] Xu Z. -Y. Kim S. Y. Hyeon D. Y. Kim D. H. Dong T. Park Y.. (2013). The *Arabidopsis* NAC transcription factor ANAC096 cooperates with bZIP-type transcription factors in dehydration and osmotic stress responses. Plant Cell 25, 4708–4724. 10.1105/tpc.113.119099, PMID: 24285786 PMC3875745

[ref59] Xu T. Purcell M. Zucchi P. Helentjaris T. Bogorad L. (2001). TRM1, a YY1-like suppressor of rbcS-m3 expression in maize mesophyll cells. Proc. Natl. Acad. Sci. 98, 2295–2300. 10.1073/pnas.041610098, PMID: 11226233 PMC30132

[ref60] Yanagisawa S. Izui K. (1990). Multiple interactions between tissue-specific nuclear proteins and the promoter of the phosphoenolpyruvate carboxylase gene for C4 photosynthesis in *Zea mays*. Mol. Gen. Genet. 224, 325–332. 10.1007/BF00262425, PMID: 2266939

[ref61] Yanagisawa S. Izui K. (1992). MNF1, a leaf tissue-specific DNA-binding protein of maize, interacts with the cauliflower mosaic virus 35S promoter as well as the C4 photosynthetic phosphoenolpyruvate carboxylase gene promoter. Plant Mol. Biol. 19, 545–553. 10.1007/BF00026781, PMID: 1627769

[ref62] Yanagisawa S. Sheen J. (1998). Involvement of maize Dof zinc finger proteins in tissue-specific and light-regulated gene expression. Plant Cell 10, 75–89. 10.1105/tpc.10.1.75, PMID: 9477573 PMC143930

