# Supplementary material for: ZmOrphan94 Transcription Factor Downregulates ZmPEPC1 Gene Expression in Maize Bundle Sheath Cells
Source: Front Plant Sci. 2021 Apr 8;12:559967. doi: 10.3389/fpls.2021.559967 (PMC8062929; doi:10.3389/fpls.2021.559967)
Supplement: Supplementary file 1 [file Data_Sheet_1.pdf]

(A)

Protein	NLS Type	Position	Aminoacid sequence
ZmOrphan94	bipartite	216	RYRKKKIKRNFGRKIKYACRKALADSQPRVRGRF
ZmCPP8	monopartite	43	IAPDPKRQRVE
ZmHB87	monopartite	254	SARKRLKKV

(B)

1 MATVLPLAAASASATPRTCISGGPVPARFLGTCMRLRIHIPPRGVACALRRRPTKYKTKIQSEEDVVAAEDVMD

chloroplast transit peptide

75 DDDDEDGALEALFKQLEEDLDNDDLSVDDNDISEEDMARFEKELAEAIEDVSGVDDSAGDSLLSSGDYGIDEQL

149 DGSERAELKTWQLRRLARALKIGRRKTSIKNLAGELGLDRGLVIEMLRNPPPKLLLMSDSLPDEVPSKSEVKEIET

225 PSTTTVDEVDTSEIKPQLELPVHVMSAEWSARKRLKKVQLETLERVYLRSKRPTNTMVSSIVQVTNLPRKTIVK

299 WFEDRREQDGVPDHRAAFKRSLSEIGASS

Figure S1. Analysis of amino acid sequences of the identified TFs. **(A)** Nuclear localisation signals (NLSs) predicted for the novel TFs using cNLS mapper. The protein sequences were derived from Grassius database (http://grassius.org/). Position refers to the first amino acid of NLS within the protein sequence in relation to first aminoacid. **(B)** Chloroplast localisation signal identified in ZmHB87 amino acid sequence using TargetP 1.1. Numbers on the left indicate ZmHB87 amino acid position.

(A) GCCAAGCCAAAAAGGAGCCTCAGCCGCAGCCGGTTCCGTT - 222 bp

5U-*ZmPEPC1-0*

GCGGTTACCGCCGAT**CACA**TGCCCAAGGCCGCGCCTTTCCGA - 180 bp

5U-*ZmPEPC1-1*

ACGCCGAGGGCCGCCCGTTCCCGTGCACAGCCACACACACAC - 140 bp

CCGCCCGCCAACGACTCCCCATCCCTATTTGAACCCACCCGCG - 95 bp

(B)

5U-*ZmPEPC1*-0	GCCAAGCCAAAAAGGAGCCTCAGCCGCAGC
5U-*ZmPEPC1*-1	GCGGTTACCGCCGAT**CACA**TGCCCAAGGCC

5U-*ZmPEPC1*-0	+	-	-	-
5U-*ZmPEPC1*-1	-	+	+	+
TRX::ZmOrphan94	+	-	-	+
TRX	-	-	+	-

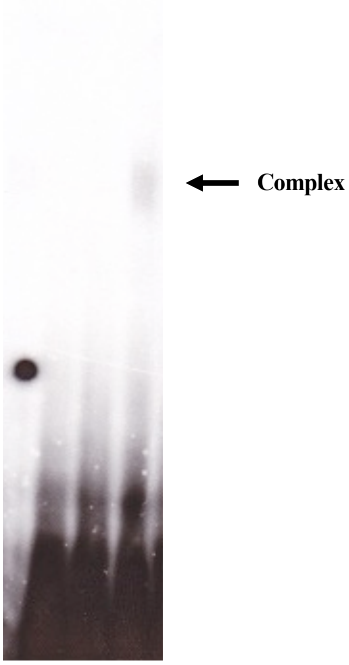

Figure S2. Analysis of the DNA- binding specificity of ZmOrphan94 to the oligonucleotide sequences with and without CACA motives derived from the 5U *ZmPEPC1* upstream region fragment. **(A)** 5U *ZmPEPC1* upstream region fragment sequence with probe sequences (underlined) used in EMSA . The binding site for ZmOrphan94 (CACA motif) is shown in bold. Base pair (bp) at the right of the panel indicate position in respect to *ZmPEPC1* ATG **(B)** EMSA representing binding specificity of TRX::ZmOrphan94 to the 32P-labeled 5U-*ZmPEPC1-1* probe containing CACA sequence. CACA motif is shown in bold.

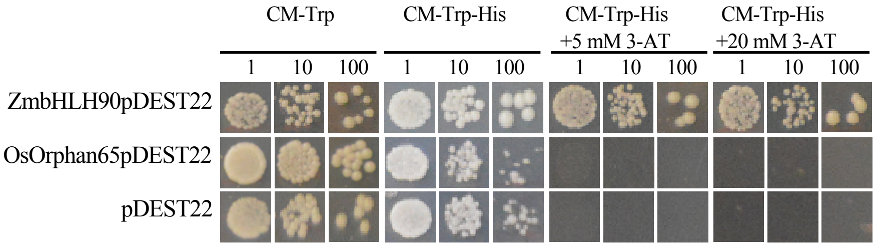

Figure S3. Analysis of the interaction between OsOrphan65 and 5U *ZmPEPC*1 upstream region. *ZmPEPC1*-5U yeast bait strain was transformed with OsOrphan65pDEST22, empty vector (EV) and ZmbHLH90pDEST22 (positive control). Growth of the transformed yeast was analysed on CM -Trp- His medium supplemented with increasing concentrations of 3-amino-1,2,4-triazole (3-AT). 1, 10 and 100 indicate yeast culture dilution factor.

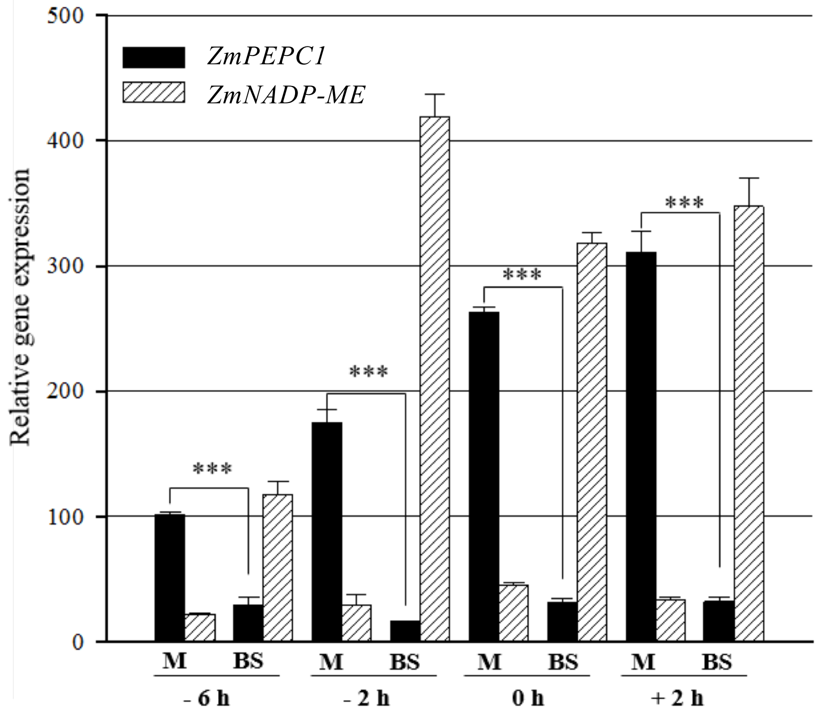

Figure S4. Analysis of *ZmPEPC*1 and *ZmNADP-ME* transcript level in mesophyll (M) and bundle sheath (BS) cells in the analysed time points. Transcript levels were analysed by RT-qPCR and normalized against the expression of two housekeeping genes (GRMZM2G144843 and GRMZM2G044552). Data represent means ± SEM (n=3). Statistical significance (T-test, ***p<0.001).

Table S1. Putative ZmCPP8 *cis*-elements identified in the F2-*ZmPEPC1* upstream region fragment and in the unrelated DNA sequence cloned into reporter vectors and used in trans-activation assays. CDE and CHR motifs are known binding sites for LIN54 TF (Schmit et al., 2009; Marceau et al., 2016). LIN54 contains the same DNA-binding motif as ZmCPP8 (CXC domain). Underlined bases indicate differences as compared with original sequence. Numbers before the elements indicate how many times a given motif was identified in the analysed sequence.

**Sequence**	**Motif**	
	**CDE (5'- CGCGG-3')**	**CHR (5'- TT(C/T)(A/G)AA-3')**
F2-*ZmPEPC1*	CGCCT,<br>CGCCA,<br>CGCCC	2 x TTTAAA
unrelated DNA	CGCGC,<br>CGCTT,<br>CGCAC,<br>CGCGT,<br>CGCTG,<br>CGCTT,	1 x TTAGAA

Table S2. List of oligonucleotides used in EMSA

Probe	Sequence (5'- 3')	Annealing temperature
5U-*ZmPEPC1*-0	ACGTGGCCAAGCCAAAAAGGAGCCTCAGCCGCAGCG	67°C
	ACATCGCTGCGGCTGAGGCTCCTTTTTGGCTTGGCC	
5U-*ZmPEPC1*-1	ACGT GGCGGTTACCGCCGATCACATGCCCAAGGCCG	67°C
	ACATCGGCCTTGGGCATGTGATCGGCGGTAACCGCC	
5U-*ZmPEPC1*-m1.1	ACGTGGCGGTTACCGCCGATTATATGCCCAAGGCCG	71°C
	ACATCGGCCTTGGGCATATAATCGGCGGTAACCGCC	
5U-*ZmPEPC1*-2	ACGTGCGAGGGCCGCCCGTTCCCGTGCACAGCCACG	71°C
	ATCACGTGGCTGTGCACGGGAACGGGCGGCCCTCGC	
5U-*ZmPEPC1*-m2.1	ACGTGCGAGGGCCGCCCGTTCCCGTGTATAGCCACG	74°C
	ATCACGTGGCTATACACGGGAACGGGCGGCCCTCGC	
5U-*ZmPEPC1*-3	ACGTGTCCCGTGCACAGCCACACACACACCCGCCCG	69°C
	ATCACGGGCGGGTGTGTGTGTGGCTGTGCACGGGAC	
5U-*ZmPEPC1*-m3.1	ACGTGTCCCGTGCACAGCTATACACACACCCGCCCG	71°C
	ATCACGGGCGGGTGTGTGTATAGCTGTGCACGGGAC	
5U-*ZmPEPC1*-m3.2	ACGTGTCCCGTGTATAGCTATACACACACCCGCCCG	68°C
	ATCACGGGCGGGTGTGTGTATAGCTATACACGGGAC	
5U-*ZmPEPC1*-m3.3	ACGTGACCCGTGTATAGCCATATATATACCCGCCCG	65°C
	ATCACGGGCGGGTATATATATGGCTATACACGGGTC	

Table S3. List of primers used in this study

Primer name	Primer sequence (5'-3')	Description
promZmPEPC1-5U	ATATTCTAGAGCCAAGCCAAAAAGGAGCCTCAGC	primer to construct baits, underlined are sequences recognized by *Xba*I enzyme
	ATATACTAGTGGCGCGGCGGGAAGCTAAGCA	primer to construct baits, underlined are sequences recognized by *Spe*I enzyme
promZmPEPC1-F1	ATATGCGGCCGCCCCTCGCCACATCCCTCCAG	primer to construct baits, underlined are sequences recognized by *Not*I enzyme
	ATATACTAGTGGGCATGTGATCGGCGGTAAC	primer to construct baits, underlined are sequences recognized by *Spe*I enzyme
promZmPEPC1-F2	CAGCGGCCGCTGCCGAGTTCCTAACCACA	primer to construct baits, underlined are sequences recognized by *Not*I enzyme
	CGACTAGTGCTTTGCAGGATGTGGAGA	primer to construct baits, underlined are sequences recognized by *Spe*I enzyme
ZmOrphan94	ATGTACGCCGACGCCCTCGC<br>CTAAGTGCTTGAATTCAGCA	primers without attB adapters to amplify ZmOrphan94
ZmCPP8	ATGGAGGCCACCCCGATCTC<br>CTAAGCCAGGTGCTGAATCTT	primers without attB adapters to amplify ZmCPP8
ZmHB87	ATGGCGACGGTGCTGCCGCTT<br>TTATGAACTAGCACCAATCTC	primers without attB adapters to amplify ZmHB87
ZmOrphan94	ATGACCTCGGAGGAGAAGGG<br>GGAGAGCGTCGTTCTGGATG	primers used in RT-qPCR
ZmActin1	GAAACCTTCGAATGCCCAGC<br>CACACCATCACCGGAATCCA	primers used in RT-qPCR
GRMZM2G044552	GGAAGCACCATCTCCCTAGC<br>ACTGCATCAAGTCCAGGAGC	primers used in RT-qPCR
GRMZM2G144843	CCGGCAATTGATGAGCCAAG<br>TGAGGATGCGAAGTGTCACC	primers used in RT-qPCR
ZmbHLH90	TTCCTTGCTGACCACCACAT<br>GAGTGGAACATCATCTGCTG	primers used in RT-qPCR
OsOrphan65	ATGTACGCCGCCATGTACC<br>CTACTTCCTTGGCTTGAGC	primers without attB adapters to amplify OsOrphan65
